# Exploring the genetic determinants underlying the differential production of an inducible chromosomal cephalosporinase - BlaB in *Yersinia enterocolitica* biotypes 1A, 1B, 2 and 4

**DOI:** 10.1038/s41598-020-67174-4

**Published:** 2020-06-23

**Authors:** Neelja Singhal, Deeksha Pandey, Nambram Somendro Singh, Manish Kumar, Jugsharan Singh Virdi

**Affiliations:** 1 Department of Microbiology, University of Delhi South Campus, New Delhi, 110021 India; 2 Department of Biophysics, University of Delhi South Campus, New Delhi, 110021 India

**Keywords:** Computational biology and bioinformatics, Microbiology, Molecular biology

## Abstract

*Yersinia enterocolitica* is an enteric bacterium which can cause severe gastroenteritis. Beta-lactams are the most widely used antibiotics against *Y. enterocolitica*. *Y. enterocolitica* produces two chromosomal β-lactamases, BlaA and BlaB. BlaB is an Ambler Class C inducible broad spectrum cephlaosporinase which showed differential enzyme activity in different biotypes of *Y. enterocolitica*. The expression of *blaB* is mainly regulated by *ampR*- the transcriptional regulator and, *ampD* - which helps in peptidoglycan recycling. The aim of this study was to identify and characterize genetic determinants underlying differential enzyme activity of BlaB in *Y. enterocolitica* biotypes 1 A, IB, 2 and 4. Thus, *ampR*, *blaB* and *ampD* were PCR-amplified and modeled *in silico*. The intercistronic region containing promoters of *ampR* and *blaB* was also investigated. Our results indicated that *blaB* was more inducible in biotypes 2 and 4, than in biotypes 1 A and 1B. Superimposition of *in silico* modeled proteins suggested that variations in amino acid sequences of AmpR, BlaB and AmpD were not responsible for hyper-production of BlaB in biotypes 2 and 4. Analysis of promoter regions of *ampR* and *blaB* revealed variations at −30, −37 and −58 positions from *blaB* transcription start site. Studies on relative expression levels of *blaB* in different biotypes by qRT-PCR indicated that nucleotide variations at these positions might contribute to a higher enzyme activity of BlaB in biotypes 2 and 4. However, this is a preliminary study and further studies including more strains of each biotype are required to strengthen our findings. Nevertheless, to the best of our knowledge, this is the first study which has investigated the genetic determinants underlying differential inducible production of BlaB in different biotypes of *Y. enterocolitica*.

## Introduction

Infections due to *Yersinia enterocolitica* have been reported from almost all the countries around the world. In developed countries, *Y. enterocolitica* ranks third among the etiological agents of bacterial gastroenteritis (after *Campylobacter* and *Salmonella*)^1^. This species comprise a heterogeneous population of bacteria divided into more than sixty serotypes and six biotypes, which show varied ecological niches, pathogenic properties and geographical distribution^2^. Strains of *Y. enterocolitica* can be classified as controversial pathogenic (biotype 1 A), highly pathogenic (biotype 1B) and less pathogenic (biotypes 2–5)^2^.

Beta-lactams are among the most widely used classes of antibiotics which are effective against several bacteria, including *Y. enterocolitica*. Bacterial resistance against this class of antibiotics has evolved primarily due to elaboration of β-lactamases, the β-lactam hydrolyzing enzymes. More than 1300 unique β-lactamases have been reported in clinical isolates^3^. Most strains of *Y. enterocolitica* produce two chromosomal β-lactamases, BlaA and BlaB^4^. BlaA is a broad-spectrum constitutively expressed Ambler class A penicillinase and BlaB is an Ambler Class C “AmpC-type” broad spectrum cephlaosporinase^5^. Chromosomal AmpC β-lactamases are usually inducible, while, except for DHA enzymes, plasmid-mediated AmpC enzymes are not^6,7^.

As reported for the AmpC enzymes present in other members of the family *Enterobacteriaceae* expression of “AmpC-type” enzymes in *Y. enterocolitica* is mainly regulated by *ampR* which is a transcriptional regulator of *ampC* and, *ampD* which participates in recycling of peptidoglycan^8^. The *ampR* located at the 5′side of *ampC* encodes a transcriptional regulator of LysR family. It is transcribed in an opposite orientation and is necessary for induction of *ampC* expression^9^. The *ampD* encodes a cytoplasmic N-acetyl-anhydromuarmoyl-L-alanine amidase that participates in recycling of peptidoglycan. AmpC β-lactamases are normally produced in bacteria at low levels (repressed state). But, during the course of medical therapy, exposure of bacteria to antibiotics like imipenem and cefoxitin results in generation and accumulation of large quantities of cell wall degradation products (muropeptides) which cannot be recycled by AmpD. The un-recycled muropeptides bind to *ampR* and inhibits its normal activity. This results in *ampC* de-repression *i.e* activation of *ampC* expression^10,11^. Thus, bacterial strains which were initially susceptible to the third generation cephalosporins, during the course of treatment develop resistance for them^12^. In *Y. enterocolitica* the *ampR-ampC* system was studied in a strain IP97, serotype O: 5b and was reportedly similar to the *ampR-ampC* system of *Citrobacter freundii*^8^.

Earlier studies have shown that *Y. enterocolitica* might produce two β-lactamases (BlaA and BlaB), only one or none of them^13–15^. Also, β-lactamases were reportedly differentially inducible in different biotypes of *Y. enterocolitica*^13,14,16^. Thus, the aim of the present study was to identify and characterize genetic determinants underlying differential inducible expression of *blaB* in *Y. enterocolitica* strains of biotypes 1 A, IB, 2 and 4. Besides modifications/mutations in the β-lactamase genes, mutations in *ampR* and *ampD* might also result in variable production of chromosomal β-lactamases^17–19^. Hence, gene sequences of *ampR*, *blaB* and *ampD* were investigated and, their 3D structures were *in silico* modeled to understand their role in differential inducible expression of *blaB* in *Y. enterocolitica* biotypes 1 A, IB, 2 and 4. Since, mutations in the promoters can also play an important role in the expression levels of β-lactamases; hence, the intercistronic region containing promoters of *ampR* and *blaB* were also investigated. To the best of our knowledge, this is the first study which has investigated the genetic determinants underlying differential inducible expression of *blaB* in *Y. enterocolitica* strains of different biotypes.

## Results

### Specific activity of BlaB before and after induction with imipenem and phenotypic detection of AmpC production

All the strains showed an increase in the β-lactamase specific activity after induction with imipenem. Strains of biotypes 1 A and 1B showed a *ca*. 2 fold increase, while of biotypes 2 and 4 showed *ca*. 5 fold increase in BlaB production after induction (Table 1). All the strains tested negative for AmpC production using AmpC E-test strips.

**Table 1:** Details of *Y. enterocolitica* strains and measurement of β-lactamase specific activity before and after induction within imipenem.

Strain	Biotype	Serotype	Country of origin	Mean specific activity of β-lactamase ± SEM (µmol/min/mg of protein)	
				Un induced	Induced
IP27433	1 A	0:6, 30-6, 31	India	0.120 ± 0.02	0.297 ± 0.01
8081	1B	0:8	USA	0.028 ± 0.02	0.049 ± 0.02
W22703	2	0:9	Europe	0.018 ± 0.01	0.091 ± 0.01
IP134	4	0:3	Europe	0.021 ± 0.01	0.095 ± 0.02

All values are represented as mean ± standard error of mean (SEM).

### PCR amplification, sequencing and multiple sequence alignment (MSA) of the inter cistronic region of *ampR* and *blaB*

**Figure 1:**
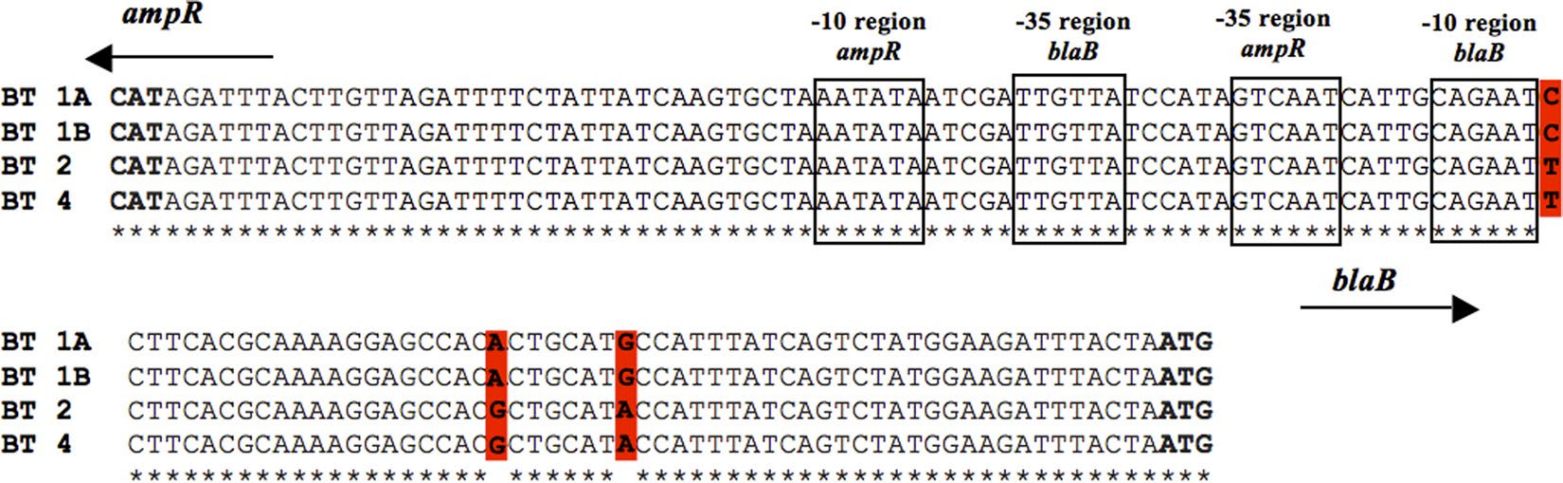
Multiple sequence alignment of the intercistronic region of *ampR and blaB* of *Y. enterocolitica* biotypes 1 A, 1B, 2 and 4. The −10 and −35 regions of *ampR* and *blaB* are enclosed in boxes. CAT and ATG (shown in bold faces) denote the transcription start site of *ampR* and *blaB*, respectively. Nucleotide variations observed at −30, −37 and −58 positions from *blaB* transcription start site are shown in red colour and in bold faces. Arrows indicate the orientation of transcription of *ampR* and *blaB*.

The primer pair B11f and B12r resulted in the desired amplicon of 1076 bp in strains of all the biotypes. BLAST analysis of the sequenced PCR amplicons confirmed that these encoded the intercistronic region of *ampR* and *blaB*, along with partial gene sequences of *ampR* and *blaB*. As expected, *blaB* and its regulator *ampR* were linked in opposite orientations. MSA of the intercistronic region containing the promoter sequences of *blaB* and *ampR* of strains of different biotypes revealed that the −10 and −35 regions of *ampR* and *blaB* promoters were similar in all biotypes. However, variations *viz*. G → A at −30, A → G at −37 and C → T at −58 positions from the *ampC* transcription start site were observed in biotypes 2 and 4 (Fig. 1).

### PCR amplification, sequencing and multiple sequence alignment (MSA) of complete coding sequence (CCDS) of ampR, *blaB* and *ampD*

Primer pairs RF and RB resulted in the desired amplicons of 730 bp, B15 and B16 in 1002 bp, DF and DR in 521 bp in strains of all the biotypes. Sequencing of the PCR amplicons followed by BLAST analysis confirmed that these encoded for AmpR, BlaB and AmpD, respectively.

Analysis of MSA of amino acid sequences of AmpR revealed that AmpR sequences were similar in all the biotypes, except for a few variations. The three critical amino acid positions of AmpR, *viz*. G-102, D-135, and Y-264, were conserved in all the strains. In *Y. enterocolitica* biotype 1B, D was replaced by H at amino acid position 82, T by A at position 103 and S by P at position 207. In *Y. enterocolitica* biotype 1 A, D was replaced by N at amino acid position 178 and, R was replaced by K at amino acid position 185. In both *Y. enterocoallitica* strains of biotypes 1 A and 1B, I was replaced by M at amino acid position 92. Amino acid sequences of AmpR of strains of biotypes 2 and 4 were identical (Table 2, Supplementary Figure 1).

**Table 2:** Details of amino acid variations in AmpR, BlaB and AmpD in different biotypes of *Y. enterocolitica*.

Protein	Biotype	Amino acid variation	Amino acid position
AmpR	1B	D → H	82
	1 A, 1B	I → M	92
	1B	T → A	103
	1 A	D → N	176
	1 A	R → K	185
	1B	S → P	207
BlaB	2, 4	Q → L	31
	1B	N → K	39
	1B	V → I	57
	1B	A → T	75
	1 A	M → I	199
	4	T → P	251
	1B	G → A	271
	1 A	E → A	277
	1 A, 1B	N → S	301
	1 A, 1B	R → G	309
AmpD	1 A, 1B	T → A	34
	1B	Q → R	55
	1B	A → G	72
	1 A, 1B	E → G	73
	1B	T → A	106
	1 A	V → A	190
	1 A, 2	S → N	145

Analysis of MSA of amino acid sequences of BlaB revealed that the sequences were similar, except for a few variations. Analysis of MSA of amino acid sequences of BlaB revealed that the sequences were similar, except for a few variations. In *Y. enterocolitica* 1B, S was replaced by A at amino acid position 14, T by S at position 22, S by at T at position 26, N by K at position 39, V by I at position 57, A by T at position 75 and G by A at position 271. In *Y. enterocolitica* biotypes 1 A and 1B, S was replaced by T at amino acid position 25, S by N at 301, R by G at 308. In biotype 1 A, A was replaced S at amino acid position 13, T by S at position 21, M by I at position 199 and E by A at position 277. In biotype 4 an aminoacid was missing at amino acid position 166 and T was replaced by P at position 251(Table 2, Supplementary Figure 2).

Analysis of MSA of amino acid sequences of AmpD revealed that amino acid sequences were similar in all the biotypes, except for a few variations. In *Y. enterocolitica* biotype 1B, Q was replaced by R at amino acid position 55, A by G at position 72 and T by A at position 106. In biotype 1 A, V was replaced by A at amino acid position 140. In both biotypes 1 A and 1B, T was replaced by A and E by G at amino acid positions 34 and 73, respectively. In *Y. enterocolitica* strains of biotypes 1 A and 2, S was replaced by N at amino acid position 145 (Table 2, Supplementary Figure 3).

### modeling of AmpR, AmpC and AmpD, evaluation and superimposition of the protein models

The top three templates (PDB ID: 5mmh_A, 5y2y, 5z50_A) which exhibited a sequence identity of more than 90% with AmpR of *Y. enterocolitica* were used as templates for modeling AmpR with I-TASSER. The C-scores of the final selected models for AmpR of *Y. enterocolitica* strains 1 A, 1B and 2/4 were, −0.78, −0.76 and −0.90, respectively. The selected models were further validated for accuracy of the prediction. The PROCHECK results indicated that more than 75% of the residues of the modeled AmpR proteins were in the allowed regions of the Ramachandran map. The average ERRAT scores and Verify-3D scores further confirmed that the predicted 3-D models were reliable and within the acceptable range. The results of the AmpR model validation are presented in Supplementary Table 1. The superimposed structure representing 3D model of the AmpR present in *Y. enterocolitica* biotypes 1 A, 1B, 2/4 is shown in Supplementary Figure 4 and the root mean square deviation (RMSD) values in Supplementary Table 2.

The top five templates (PDB ID: 2zc7_A, 1ga0_A, 5ggw_B) which exhibited a sequence identity of more than 90% with BlaB of *Y. enterocolitica* were used as templates for modeling BlaB with I-TASSER. The C-scores of the final selected models for BlaB of *Y. enterocolitica* biotypes 1 A, 1B, 2 and 4 were −0.73,−1.06, −0.16 and −1.86, respectively. The results of the BlaB model validation are presented in Supplementary Table 3. The superimposed structure representing 3D model of the BlaB present in *Y. enterocolitica* strains of biotypes 1 A, 1B, 2 and 4 is shown in Supplementary Figure 5 and the RMSD values in Supplementary Table 4.

The top three templates (PDB ID: 6fhg_A, 1j3g_A, 6j3w_A) which exhibited a sequence identity of more than 90% with AmpD of *Y. enterocolitica* were used as templates for modeling AmpD. The C scores of the final selected models for AmpD of *Y. enterocolitica* biotypes 1 A, 1B, 2 and 4 were −1.13, −1.59, −1.39 and −1.162, respectively. The results of the AmpD model validation are presented in Supplementary Table 5. The superimposed structure representing the 3D model of AmpD in *Y. enterocolitica* biotypes 1 A, 1B, 2 and 4 is shown in Supplementary Figure 6 and the RMSD values in Supplementary Table 6.

### Relative expression of *ampR* and *blaB* as determined by qRT–PCR

**Figure 2:**
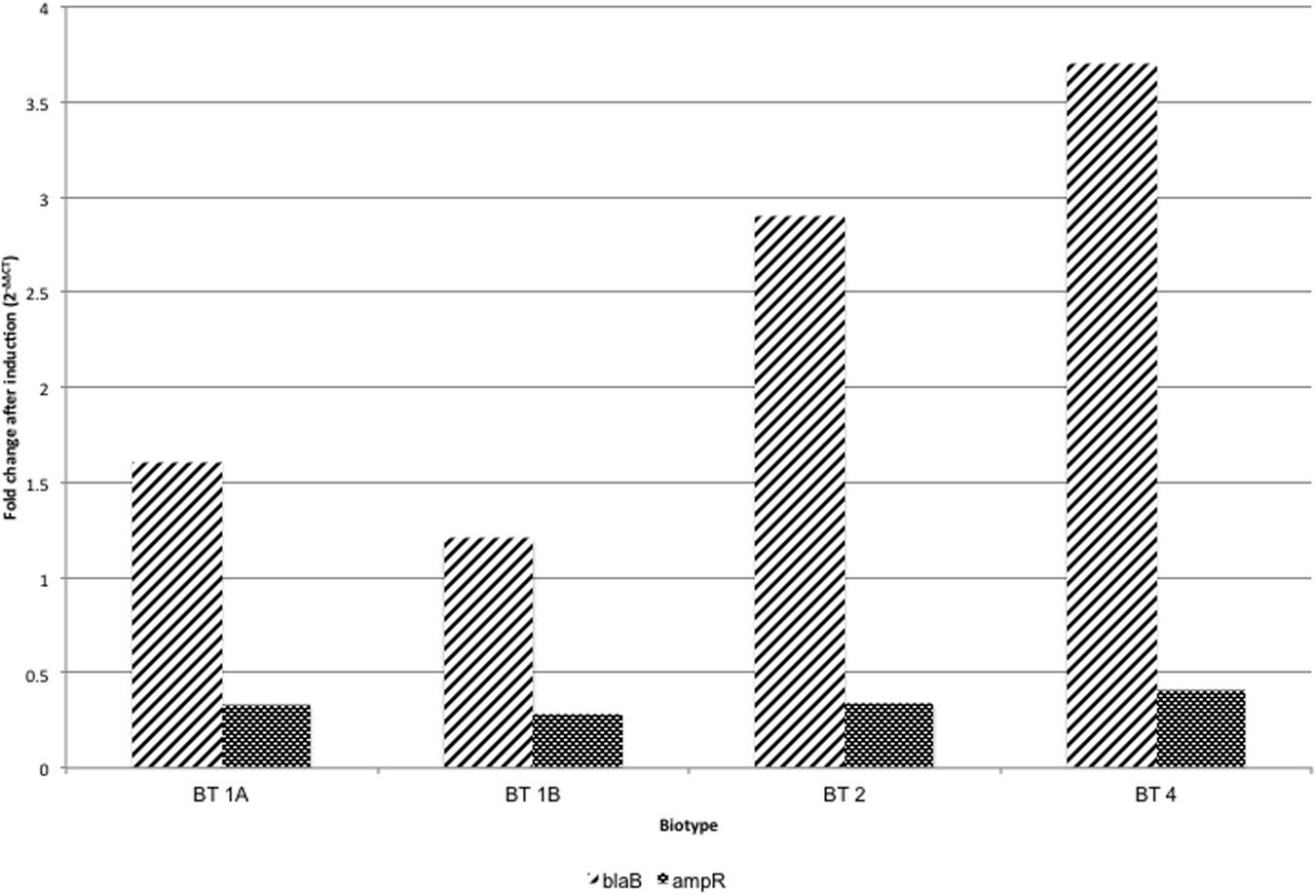
Fold change in mRNA expression levels of *ampR* and *blaB* after induction with imipenem.

The relative change in expression levels of mRNA of *ampR* after induction was observed to be non significant in all the strains. However, all the strains showed an increase in the expression levels of mRNA of *blaB* after induction. The fold change in relative expression of *blaB* was more in strains of biotypes 2 and 4 (~3–4 times) than in biotypes 1 A and 1B (~1.3 times) (Fig. 2).

## Discussion

The aim of the present study was to understand reasons underlying differential inducible production of BlaB, an “AmpC-type” β-lactamase in *Y. enterocolitica* strains of biotypes 1 A, 1B, 2 and 4. Our results indicated that BlaB was inducible in strains of biotypes 1 A, 1B, 2 and 4. Interestingly, the level of production of BlaB after induction was more in biotypes 2 and 4 (~5 times) than in biotypes 1 A and 1B (~2 times). Several studies have reported that the level of production of BlaB after induction varied among different biotypes of *Y. enterocolitica*^13,14^. Another interesting observation was that the AmpC E-test strips failed to detect BlaB production, while the spectrophotometric enzyme assays and PCR-amplification confirmed that “AmpC-type” inducible cephalosporinases were present in *Y. enterocolitica* biotypes 1 A, 1B, 2 and 4. Thus, our results suggest that E-test strips of cefotetan/cefotetan+cloxacillin should not be used for phenotypic detection of AmpC production in *Y. enterocolitica*.

It was reported that increase in the level of β-lactamase activity in *Y. enterocolitica* when cultivated in the growth medium containing imipenem indicated that production of these enzymes might be subject to regulatory control^20^. There can be several reasons underlying variations in the expression/production of chromosomal β-lactamase like, mutations in the gene and/or promoter regions, modifications in the regulatory regions^19^ *etc*. Mutations in *ampR* - the transcriptional regulator of *ampC* are less frequent, but might also result in hyperinducibility or constitutive hyperproduction of AmpC^17^. In clinical isolates, mutations in *ampD* were frequently associated with hyperinducibility or hyperproduction of AmpC^18^. Thus, genes encoding AmpR, BlaB and AmpD including the intercistronic region of *ampR* and *blaB* were PCR-amplified, compared and analyzed in *Y. enterocolitica* strains of biotypes 1 A, 1B, 2 and 4.

Analysis of AmpR sequences of biotypes 1 A, 1B, 2 and 4 revealed that two domains were present - a substrate binding domain of LysR-type transcriptional regulators (LTTRs) of PBP2_LTTR_substrate super family (accession- cl25412) and, a domain of bacterial regulatory helix-turn-helix protein, LysR family - HTH_1 (pfam00126). Also, the three amino acid positions *viz*. G-102, D-135, and Y-264 that are important for the biological activity of AmpR were found to be conserved in AmpR of strains of all biotypes^8^. MSA analysis revealed that the AmpR sequence of biotypes 2 and 4 were identical, while a few variations were present in AmpR sequences of other biotypes. Hence, AmpR of different biotypes were *in silico* modeled and superimposed. The 3D protein models of AmpR variants showed a strong structural alignment and significantly lower RMSD values. This indicated that variations in AmpR sequences might not be responsible for differential inducible production of BlaB in different biotypes.

Analysis of amino acid sequences of BlaB revealed that the two significant motifs conserved in the Ambler class C β-lactamases - ^151^SXXK^154^ and ^342^KTG^344^, (X can be any amino acid) were conserved in BlaB of all the biotypes. Chen *et al*.^21^ reported that the key catalytic residues of the AmpC enzymes are: S-64, K-67, Y-150, N-152, K-315 and A-318 and, substitutions at these sites decreased the enzymatic activity of AmpC. Though, all the key catalytic residues were found to be present in BlaB of biotypes 1 A, 1B, 2 and 4 but their respective positions were S-89, K-92, Y-175, N-177, K-342 and A-345. The 24 amino acids long signal peptide at the N-terminal was excluded from comparative analysis. Though, no variations were observed in the catalytic residues, a few variations were observed at some other sites. The results of *in silico* protein modeling and superimposition of 3D models of BlaB variants revealed a strong structural alignment and significantly lower RMSD values. This indicated that variations in amino acid sequences of BlaB might not be responsible for differential inducible production BlaB in different biotypes.

The critical amino acid residue positions in *Y. enterocolitica* AmpD are A-43,H-123H (the amidase catalytic sites), H-123, D-133 (Zn binding sites), and K-131, D-133, A-43, V-57, W-64 (substrate binding sites)^22^. Though, no variations were observed at these sites, a few variations were observed at some other positions. The results of the superimposition of the 3D protein models of AmpD variants indicated that variations in AmpD sequences might not be responsible for differential inducibility of *blaB* in different biotypes.

Mutations in the promoter sequences and/or insertions in the promoter regions have been reportedly associated with hyper production of β-lactamases in the family *Enterobacteriaceae*^23^. Hence, the intercistronic region between the start codons of *ampR* and *blaB* were investigated for their role in differential regulation of expression of *blaB*. The intercistronic region between *ampR* and *blaB* start codons, known as the control region is 135 bp long and, contains promoters for both and, the AmpR binding site^8^. A previous study reported that the −35 and −10 regions of the promoter*s of ampR* and *ampC* of *Y. enterocolitica* were similar to the *ampR* and *ampC* promoters of *Citrobacter freundii*^8,24^. Our results indicated that the sequence of the *ampR* promoters including the −35 and −10 regions were identical in all biotypes. Hence, these might not be responsible for higher inducible expression of *blaB* in biotypes 2 and 4. Results of the MSA of *blaB* promoters revealed that the −35 and −10 regions of the *blaB* promoter*s* were identical in all biotypes. However, variations *viz*. G → A at −30, A → G at −37, C → T at −58 positions from the *ampC* transcription start site were observed in biotypes 2 and 4. Several researchers have reported that in *Enterobacteriaceae*, mutations and insertions at sites other than the −35 (Pribnow box) and −10 region (TATA box) of the *ampC* promoters resulted in a hyper expression of *ampC* in clinical isolates^23^^,^^25–28^.

To validate the role of these variations, if any, in differential inducible expression of *blaB* in strains of biotypes 2 and 4, expression levels of *ampR* and *blaB* were measured before and after induction using qRT-PCR. The relative expression levels of mRNA of *ampR* after induction were found to be non-significant in all biotypes. This might be attributed to the fact that the *ampR* promoter regions were conserved in all biotypes. However, the difference in the relative expression levels of mRNA of *blaB* after induction was significant. The fold change in relative expression of *blaB* in strains of biotypes 2 and 4 was *ca*. 3–4 times, while it was *ca*. 1.3 times in biotypes 1 A and 1B, which broadly reiterated the results obtained by measurement of β-lactamase specific activity. This suggested that variations in the promoter regions might be responsible for higher inducible production of BlaB which was observed in strains of biotypes 2 and 4. It is pertinent to mention here that though, the fold changes (2^–∆∆CT^) in mRNA levels of *blaB* were similar, these were not identical with the fold changes observed in the β-lactamase specific activity after induction. Such small variations in the protein production and mRNA expression studies might be attributed to the differences in the post-translational modifications and post-transcriptional processing of proteins and mRNAs, respectively. Also, the differences in the degradation rates of proteins and mRNA during bacterial growth might also contribute to similar, but non-identical levels of bacterial mRNA and proteins.

To summarize, our results indicated that *blaB* was more inducible in biotypes 2 and 4, than in biotypes 1 A and 1B. Though, a few variations were present in amino acid sequences of AmpR, BlaB and AmpD, superimposition of the 3D protein models of AmpR, BlaB and AmpD suggested that these variations were not responsible for hyper production of BlaB in biotypes 2 and 4. Analysis of the promoter regions of *ampR* and *blaB* revealed variations at −30, −37 and −58 regions of the *blaB* promoter. Studies on relative expression levels of *blaB* in different biotypes by qRT-PCR suggested that nucleotide variations at these positions might be important for higher levels of transcription and, consequently a higher enzyme activity in biotypes 2 and 4, after induction. The results of this study are expected to help in devising novel intervention strategies against yersiniosis. However, this is a preliminary study and further experiments on promoter strength incorporating more strains of each biotype are required to strengthen these findings.

## Materials and Methods

### Bacterial strains

In the present study, four clinical strains of *Y. enterocolitica* representing biotypes 1 A, 1B, 2 and 4 were used. *Y. enterocolitica* strain representing biotype 1 A was isolated from India, while strains of biotypes 1B, 2 and 4 were isolated from different parts of the world and were received as kind gifts from foreign laboratories. The details of these strains *viz*. serotypes, laboratory and reference laboratory accession numbers and country of origin are given in Table 1. All the strains were maintained on tryptone soy agar at 4 °C.

### Induction of *blaB* expression, preparation of cell lysates and spectrophotometeric assay of β -lactamases

*Y. enterocolitica* strains were induced for production of BlaB by cultivating bacteria in tryptic soy broth (TSB) containing imipenem (concentration − 0.5 mg/l). The methods for induction and preparation of cell lysates have been described earlier^15^. The enzyme activity of BlaB was assessed spectrophotometrically by hydrolysis of nitrocefin. The contents of the assay mixture and the methods have been described earlier^15,22^. The enzyme specific activity was expressed as µmol of nitrocefin hydrolyzed/min/mg of protein. The experiments were repeated for each strain in triplicates and the average results were reported ± standard error mean (SEM).

### Phenotypic detection of AmpC production using E-test strips

Phenotypic detection of AmpC production after induction with imipenem was done using E-test strips of cefotetan/cefotetan+cloxacillin (bioMerieux Inc., MO, USA) following the methods described earlier^29^. Following AmpC E-test, if cefotetan/cefotetan + cloxacillin (CN/CNI) ration was ≥8 a strain was considered as AmpC producer.

### Isolation of genomic DNA

The genomic DNA was isolated from overnight grown bacterial culture in TSB at 28 °C. The total genomic DNA was isolated using DNeasy Tissue kit (Qiagen, Hilden, Germany), eluted in sterile water and quantitated spectrophotometrically at 260 nm.

### PCR amplification of the intercistronic region containing promoters of *blaB* and *ampR* and, complete coding sequences (CCDS) of *ampR*, *blaB* and *ampD*

The intercistronic region containing promoters of *ampR* and *blaB* along with the partial gene regions of *ampR* and *blaB* was amplified using primers B11f and B12r. Primer pairs RF and RB were used for amplification of CCDS of *ampR*, B15 and B16 for amplification of CCDS of *blaB*, and DF and DR for amplification of CCDS of *ampD*. The components of the PCR reaction mixture and PCR conditions, except the annealing temperatures have been described earlier^30^. The details of the primer sequences and the annealing temperatures are presented in Table 3. The PCR amplicons were electrophoresed and visualized under UV transilluminator.

**Table 3:** Details of primers used for amplification of intercistronic region containing promoters of *ampR* and *blaB* and, CCDS of *ampR, blaB and ampD* in different biotypes of *Y. enterocolitica*.

Primer name	Primer sequence	Gene	Amplicon size (bp)	Reference
B11f and B12r	F:5′CCTGACTTTTTCACGTATTAT3′ R:5′GGGGATAGTGATAAAGGTAT3′	intercistronic region of *ampR* and *blaB* and partial regions of *ampR* and *blab*	1076	22
RF and RB	F:5′CTTTATTCGTATTTCACGCG 3′R:5′CTATTCTCCCTCAGACTTCA 3′	*ampR*	730	22
B15F and B16R	F: 5′TGACGGAAAGCCGCAATTCT3′R:5′TCATAGAAGCGTCAACGCAA3′	*BlaB*	1002	29
DF and DR	F:5′GCCAGAAGGTGAAGCTCCTT3′R:5′CTCTGGTTAATACTGCATGA3’	*ampD*	521	36

### Sequencing of the intercistronic regions of *ampR* and *blaB* and, CCDS of *ampR*, *blaB* and *ampD*

PCR amplicons containing intercistronic regions of *ampR* and *blaB* along with their promoter regions and, CCDS of *ampR*, *blaB* and *ampD* were purified and sequenced at a commercial facility (Invitrogen BioServices India Pvt. Ltd., Bangalore, India). The sequences were identified by homology search using NCBI-BLAST (https://blast.ncbi.nlm.nih.gov/Blast.cgi). The GenBank accession number of *ampR* of *Y. enterocolitica* strains 20, 8081, IP134, W22703 were MK511112, MN242777- MN242779 respectively, of *blaB* of *Y. enterocolitica* strains 20, 8081, IP134, W22703 were MN172161- MN172164 respectively and, of *ampD* of strains 20, 8081, IP134, W22703 were MK511120, MN172158-MN172160 respectively. The CCDS of *ampR*, *blaB* and *ampD* were translated using ExPASy (https://www.expasy.org/) and aligned by Clustal Omega (http://www.ebi.uc.ak/clustal).

### 3-D structure predictions of AmpR, BlaB and AmpD: modeling and validation

Since the protein structures of AmpR, BlaB and AmpD of *Y. enterocoloitica* are not known; the 3D structures of AmpR, BlaB and AmpD of strains of different biotypes were predicted using the web interface iterative threading assembly refinement (I-TASSER) *(*https://zhanglab.ccmb.med.umich.edu/I-TASSER/*)*. Since the amino acid sequences of AmpR of strains of biotype 2 and 4 were identical, hence only one sequence representing AmpR of both the biotypes were modeled. Five models were predicted by I-TASSER for AmpR, BlaB and AmpD each, of which the best model was selected on the basis of the confidence score (C-score). The selected models were further validated for accuracy using the programs PROCHECK^31^ ERRAT^32^ and Verify 3D^33^. The protein models of AmpR, BlaB and AmpD of each biotype were superimposed and visualized using PyMol (https://pymol.org/2/).

### Determination of expression levels of *ampR* and *blaB* by real time PCR (qRT-PCR)

The effect of promoter variations on relative expression levels of *ampR* and *blaB* in *Y. enterocolitica* strains before and after induction with imipenem was studied by qRT-PCR. Total RNA was extracted from the bacterial cultures before and after induction with imipenem using SV Total RNA isolation system (Promega, Madison, WI, USA). The concentration of RNA was quantified spectrophotometrically. The cDNA was prepared from each sample (template −1 μg RNA) using a commercial kit (cDNA synthesis kit, TaKaRa, Shiga, Japan). Primers were designed for amplification of *ampR* and *blaB* using the software Primer3 (http://simgene.com/Primer3). One of the housekeeping genes, *gapA* was included as the reference gene^34^. The details of the primers are given in the Supplementary Table 7. The qRT-PCR was performed at a commercial facility (Genotypic Technology Pvt. Ltd. Bengaluru, India) using SYBR Green chemistry (Brilliant II SYBR Green qPCR master mix, Agilent Technologies, USA) in Stratagene mx3005P instrument (Agilent Technologies, USA). The cycling conditions for amplification were as follows: initial denaturation for 95 °C for 10 min followed by 40 cycles at 95 °C for 30 sec, 58 °C for 30 sec. The mean Ct value of technical replicates was used to calculate the relative expression level of genes. The experiments were performed in triplicate and the average results were reported ± SD. The relative quantification of genes was performed using the standard 2^−ΔΔCt^ method, also known as the delta-delta CT method, as described by Pfaffl^35,36^.

## Supplementary information

Supplementary information.

Supplementary information2.

Supplementary information3.

Supplementary information4.

Supplementary information5.

Supplementary information6.

Supplementary information7.
