# Supplementary figures and images for: Exploring the genetic determinants underlying the differential production of an inducible chromosomal cephalosporinase - BlaB in Yersinia enterocolitica biotypes 1A, 1B, 2 and 4
Source: Sci Rep. 2020 Jun 23;10:10167. doi: 10.1038/s41598-020-67174-4 (PMC7311522; doi:10.1038/s41598-020-67174-4)

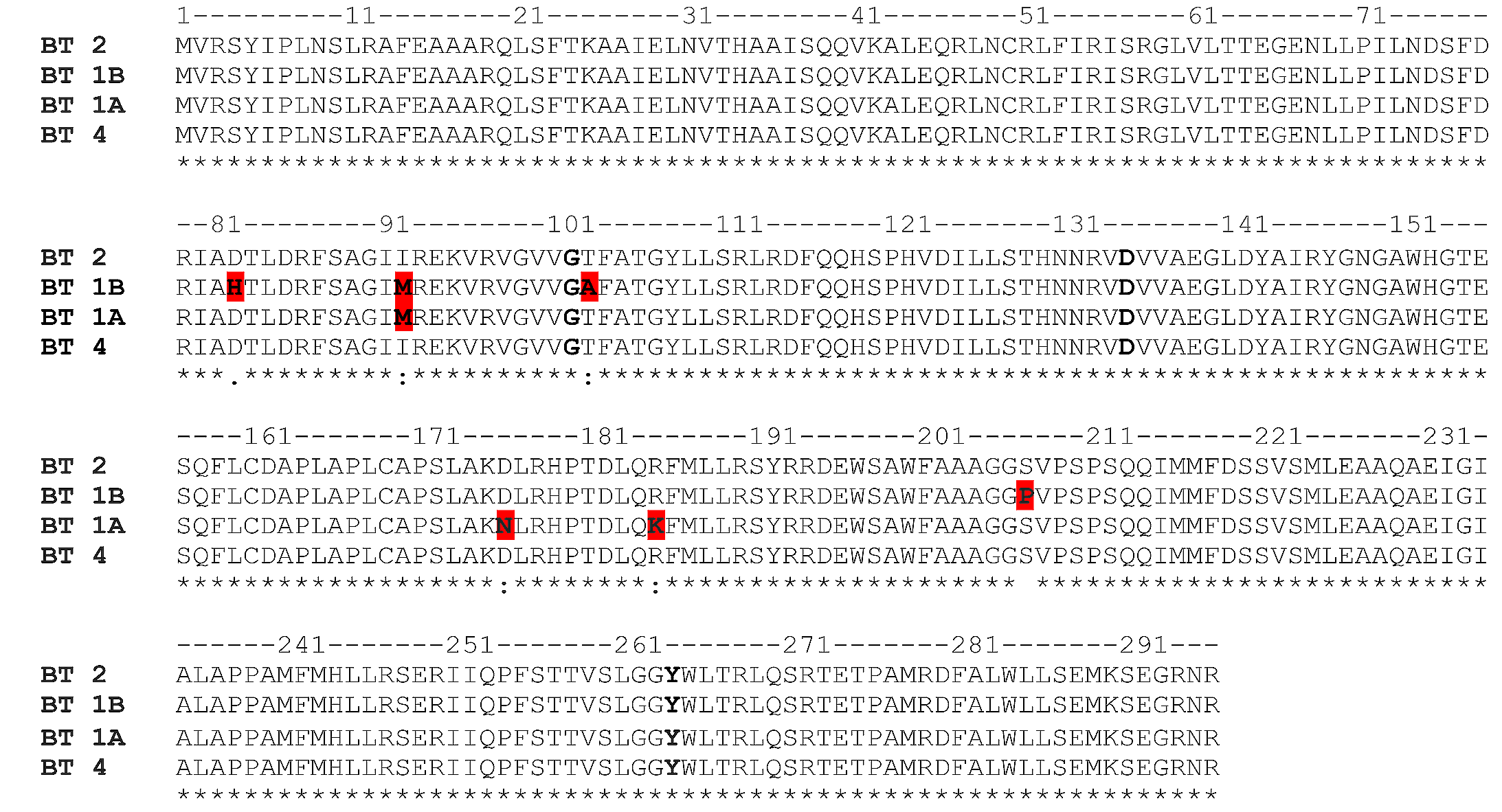

Supplement: Supplementary file 1 — Supplementary information. [file 41598_2020_67174_MOESM1_ESM.tif]

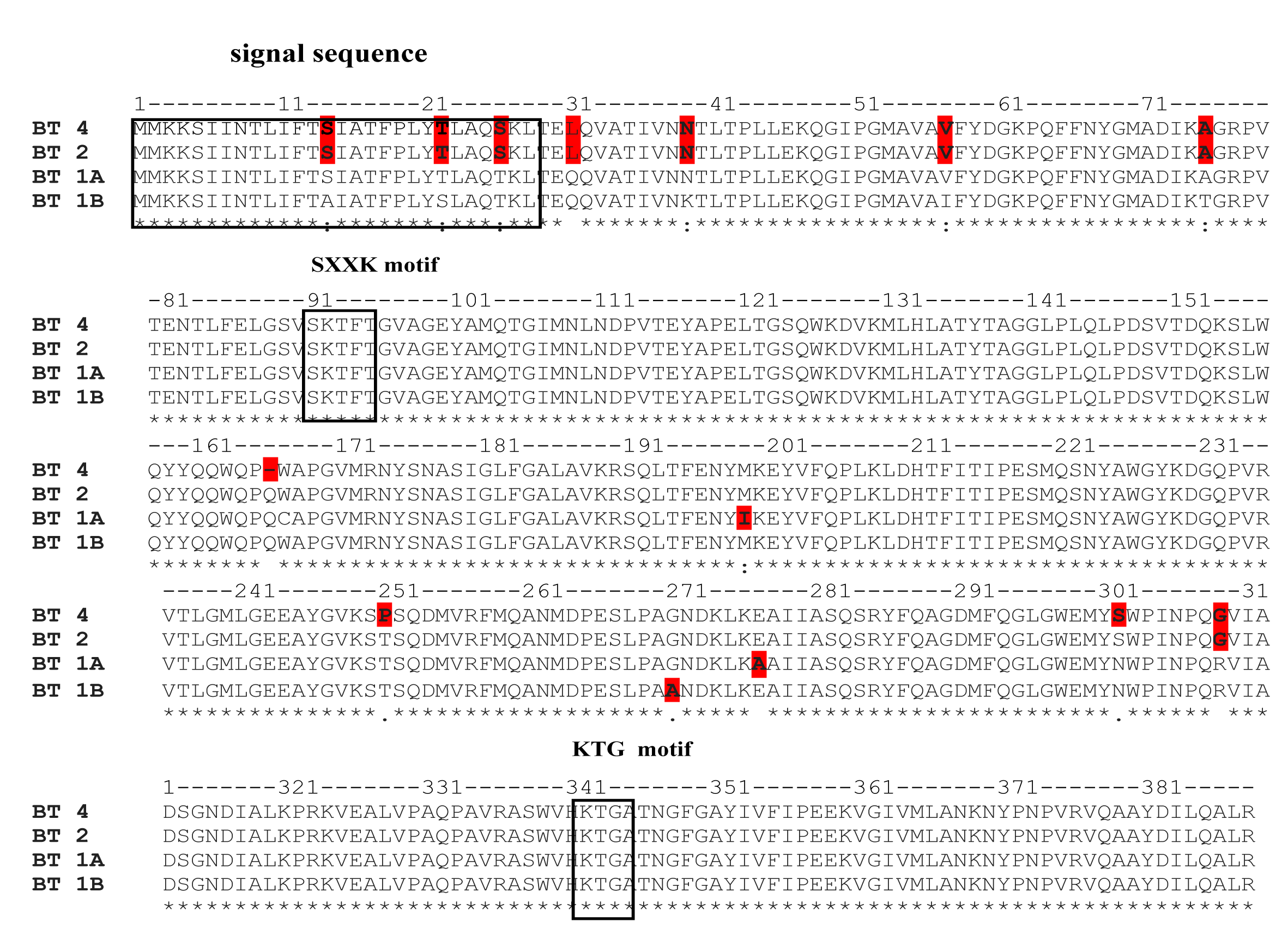

Supplement: Supplementary file 2 — Supplementary information2. [file 41598_2020_67174_MOESM2_ESM.tif]

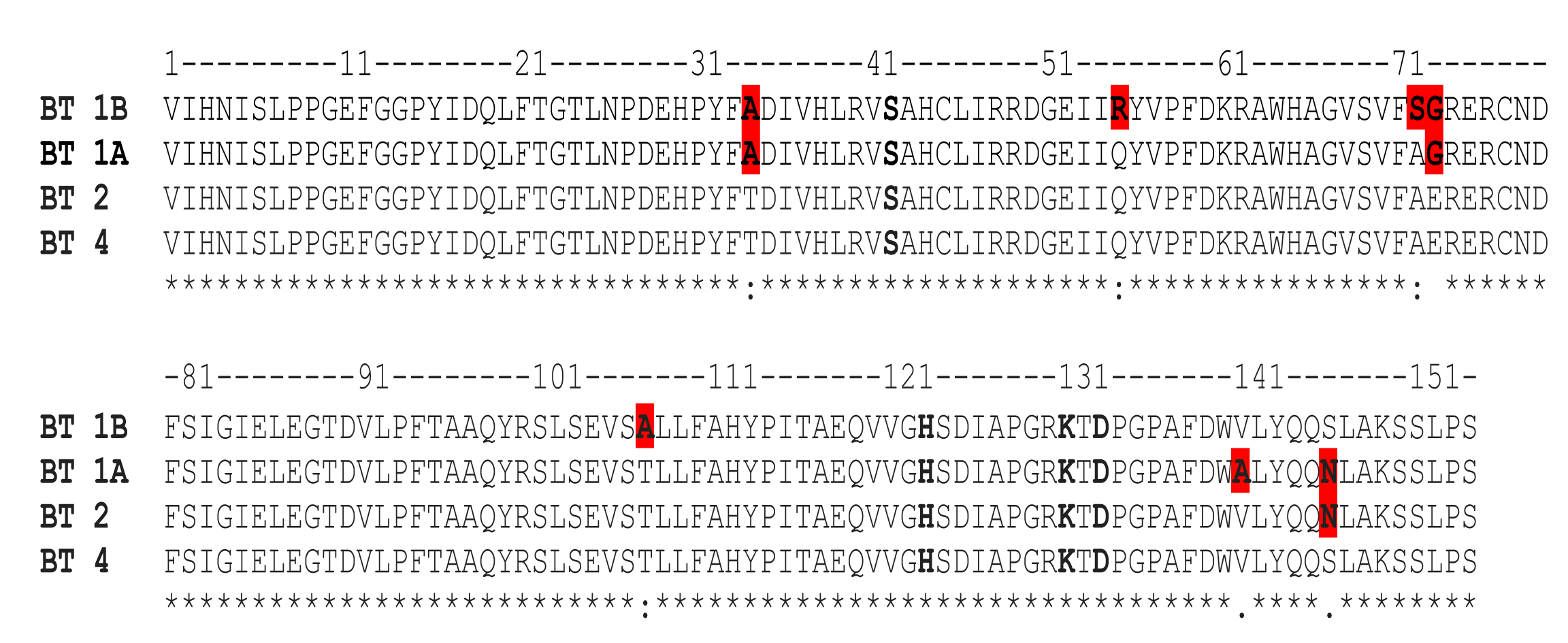

Supplement: Supplementary file 3 — Supplementary information3. [file 41598_2020_67174_MOESM3_ESM.tif]

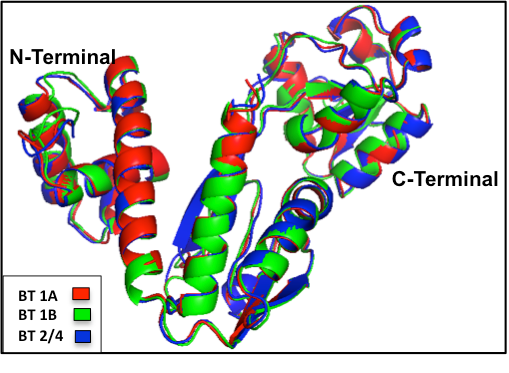

Supplement: Supplementary file 4 — Supplementary information4. [file 41598_2020_67174_MOESM4_ESM.tiff]

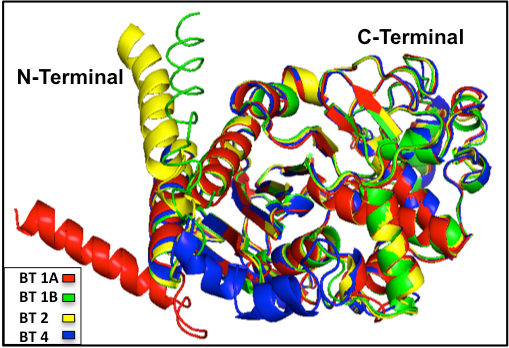

Supplement: Supplementary file 5 — Supplementary information5. [file 41598_2020_67174_MOESM5_ESM.tiff]

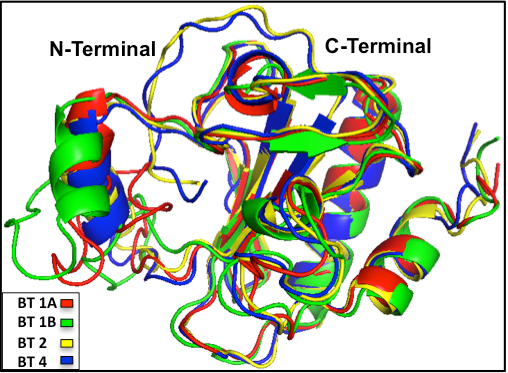

Supplement: Supplementary file 6 — Supplementary information6. [file 41598_2020_67174_MOESM6_ESM.tiff]
