# Supplementary material for: Exploring the genetic determinants underlying the differential production of an inducible chromosomal cephalosporinase - BlaB in Yersinia enterocolitica biotypes 1A, 1B, 2 and 4
Source: Sci Rep. 2020 Jun 23;10:10167. doi: 10.1038/s41598-020-67174-4 (PMC7311522; doi:10.1038/s41598-020-67174-4)
Supplement: Supplementary file 7 — Supplementary information7. [file 41598_2020_67174_MOESM7_ESM.docx]

**Supplementary figure legends**

**Supplementary Figure 1.** Multiple sequence alignment of AmpR sequences present in *Y. enterocolitica* biotypes 1A, 1B, 2 and 4. The critical amino acids required for biological activity of AmpR, G-102, D-135, and Y-264 are marked in bold faces and amino acid variations are shown in red colour.

**Supplementary Figure 2.** Multiple sequence alignment of BlaB sequences present in *Y. enterocolitica* biotypes 1A, 1B, 2 and 4. The signal sequences, SXXK and KTG motifs are enclosed in boxes and amino acid variations are shown in red colour.

**Supplementary Figure 3.** Multiple sequence alignment of AmpD present in *Y. enterocolitica* biotypes 1A, 1B, 2 and 4. The amidase catalytic sites are shown in bold face and amino acid variations are shown in red colour.

**Supplementary Figure 4.** Superimposed 3D models of AmpR variants present in different biotypes of *Y. enterocolitica* (created using the software I-TASSER; https://zhanglab.ccmb.med.umich.edu/I-TASSER/)

**Supplementary Figure 5.** Superimposed 3D models of BlaB variants present in different biotypes of *Y. enterocolitica* (created using the software I-TASSER; https://zhanglab.ccmb.med.umich.edu/I-TASSER/)

**Supplementary Figure 6.** Superimposed 3D models of AmpD variants present in different biotypes of *Y. enterocolitica* (created using the software I-TASSER; https://zhanglab.ccmb.med.umich.edu/I-TASSER/)

**Table 1** Details of *Y. enterocolitica* strains and measurement of β-lactamase specific activity before and after induction within imipenem

| **Strain** | **Biotype** | **Serotype** | **Country**  **of origin** | **Mean specific activity of** **β-lactamase ± SEM**  **(µmol/min/mg of protein)** | |
| --- | --- | --- | --- | --- | --- |
|  |  |  |  | **Un induced** | **Induced** |
| IP27433 | 1A | O:6,30-6,31 | India | .120 ± .02 | .297 ± .01 |
| 8081 | 1B | O:8 | USA | .028 ± .02 | .049 ± .02 |
| W22703 | 2 | O:9 | Europe | .018 ± .01 | .091 ± .01 |
| IP134 | 4 | O:3 | Europe | .021 ± .01 | .095 ± .02 |

All values are represented as mean ± standard error of mean (SEM)

**Table 2** Details of amino acid variations in AmpR, BlaB and AmpD in different biotypes of *Y. enterocolitica*

| **Protein** | **Biotype** | **Amino acid variation** | **Amino acid position** |
| --- | --- | --- | --- |
| AmpR | 1B | D→H | 82 |
|  | 1A, 1B | I→M | 92 |
|  | 1B | T→A | 103 |
|  | 1A | D→N | 176 |
|  | 1A | R→K | 185 |
|  | 1B | S→P | 207 |
| BlaB | 2, 4 | Q→L | 31 |
|  | 1B | N→K | 39 |
|  | 1B | V→I | 57 |
|  | 1B | A→T | 75 |
|  | 1A | M→I | 199 |
|  | 4 | T→P | 251 |
|  | 1B | G→A | 271 |
|  | 1A | E→A | 277 |
|  | 1A, 1B | N→S | 301 |
|  | 1A, 1B | R→G | 309 |
| AmpD | 1A, 1B | T→A | 34 |
|  | 1B | Q→R | 55 |
|  | 1B | A→G | 72 |
|  | 1A, 1B | E→G | 73 |
|  | 1B | T→A | 106 |
|  | 1A | V→A | 190 |
|  | 1A, 2 | S→N | 145 |

**Table 3** Details of primers used for amplification of intercistronic region containing promoters of *ampR* and *blaB* and, CCDS of *ampR, blaB and ampD* in different biotypes of *Y. enterocolitica*

| **Primer name** | **Primer sequence** | **Gene** | **Amplicon size (bp)** | **Reference** |
| --- | --- | --- | --- | --- |
| B11f and B12r | F:5’CCTGACTTTTTCACGTATTAT3’ R:5’GGGGATAGTGATAAAGGTAT3’ | intercistronic region of *ampR*  and *blaB* and partial regions  of *ampR* and *blab* | 1076 | 22 |
| RF and RB | F:5’CTTTATTCGTATTTCACGCG 3’  R:5’CTATTCTCCCTCAGACTTCA 3’ | *ampR* | 730 | 22 |
| B15F and B16R | F: 5’TGACGGAAAGCCGCAATTCT3’  R:5’TCATAGAAGCGTCAACGCAA3’ | *BlaB* | 1002 | 29 |
| DF and DR | F:5’GCCAGAAGGTGAAGCTCCTT3’  R:5’CTCTGGTTAATACTGCATGA3’ | *ampD* | 521 | 36 |

**Supplementary Table 1** 3D protein model validation scores of AmpR variants present in *Y. enterocolitica* biotypes 1A, 1B, 2 and 4

| **AmpR model** | **Ramachandran plot statistics** | | | | **ERRAT Statistics** | **Verify 3D**  **Score** |
| --- | --- | --- | --- | --- | --- | --- |
|  | **Residues in most favourable region** | **Residues in additional allowed region** | **Residues in generously allowed region** | **Residues in disallowed region** |  |  |
| **BT 1A** | 77.4% | 18.1% | 2.3% | 2.3% | 84.37 | 86.82% |
| **BT 1B** | 76.5% | 17.0% | 4.2% | 2.3% | 84.66 | 79.73% |
| **BT 2/4** | 77.7% | 17.7% | 1.5% | 3.0% | 83.33 | 93.92% |

**Supplementary Table 2** Root Mean Square Deviation values of predicted 3D models of AmpR variants present in different biotypes of *Y. entercolitica*

| **AmpR model** | **BT 1B** | **BT 2/4** |
| --- | --- | --- |
| **BT 1A** | 0.498 | 0.475 |
| **BT 2/4** | 0.554 | - |

**Supplementary Table 3** 3D protein model validation scores of BlaB variants present in *Y. enterocolitica* biotypes 1A, 1B, 2 and 4

| **BlaB**  **Variants** | **Ramachandran plot statistics** | | | | **ERRAT statistics** | **Verify 3D**  **score** |
| --- | --- | --- | --- | --- | --- | --- |
|  | **Residues in most favourable region** | **Residues in additional allowed region** | **Residues in generously allowed region** | **Residues in disallowed region** |  |  |
| **BT 1A** | 84.1% | 13.5% | 1.8% | 0.6% | 89.73 | 89.95% |
| **BT 1B** | 82.6% | 15.0% | 1.5% | 0.9% | 95.26 | 91.24% |
| **BT 2** | 85.5% | 12.7% | 0.9% | 0.9% | 94.45 | 91.47% |
| **BT 4** | 83.3% | 12.9% | 2.5% | 1.3% | 88.01 | 82.13% |

**Supplementary Table 4** Root Mean Square Deviation values of predicted 3D models of BlaB variants present in different biotypes of *Y. enterocolitica*

| **BlaB modelB** | **BT 1B** | **BT 2** | **BT 4** |
| --- | --- | --- | --- |
| **BT 1A** | 0.401 | 0.387 | 0.356 |
| **BT1B** | - | 0.121 | 0.252 |
| **BT 4** | 0.252 | 0.228 | - |

**Supplementary Table 5** 3D protein model validation scores of AmpD variants present in *Y. enterocolitica* biotypes 1A, 1B, 2 and 4

| **AmpD variants** | **Ramachandran Plot Statistics** | | | | **ERRAT statistics** | **Verify 3D**  **score** |
| --- | --- | --- | --- | --- | --- | --- |
|  | **Residues in most favourable region** | **Residues in additional allowed region** | **Residues in generously allowed region** | **Residue in disallowed region** |  |  |
| **BT 1A** | 75% | 20.3% | 1.6% | 3.1% | 43.83 | 95.45% |
| **BT 1B** | 70.3% | 23.4% | 3.9% | 2.3% | 88.35 | 66.88% |
| **BT 2** | 71.3% | 21.7% | 3.1% | 3.9% | 76.02 | 98.70% |
| **BT 4** | 68.2% | 19.4% | 6.2% | 6.2% | 72.60 | 91.56% |

**Supplementary Table 6** Root Mean Square Deviation values of predicted 3D models of AmpD variants present in different biotypes of *Y. enterocolitica*

| **AmpD models** | **BT 1B** | **BT 2** | **BT 4** |
| --- | --- | --- | --- |
| **BT 1A** | 0.88 | 0.80 | 0.85 |
| **BT 1B** | - | 1.14 | 1.14 |
| **BT 4** | 1.14 | 0.40 | - |

**Supplementary Table 7** Details of primers used for analysis of expression of *ampR* and *blaB* using qRT-PCR before and after induction with imipenem

| **Gene** | **Primer sequence** | **Amplicon size** | **Reference** |
| --- | --- | --- | --- |
| *blaB* | F:5'AATCACGTTATTTCCAGG3'  R: 5'AAAGCCATTGGTTGCACC3' | 200 bp | this study |
| *ampR* | F: 5'TTACTAAGGCTGCTATCG3'  R: 5'GTATGCGCGATAGATCA3' | 181 bp | this study |
| *gapA* | F:5’TAGGTATCAACGGTTTCGGC3'  R:5’ACAGTACCGTCGAAACGACC3' | 160 bp | 34 |
